# Circulating Cytokines in Melanoma Prognosis: Current Evidence and Future Perspectives

**DOI:** 10.3390/medicina62050960

**Published:** 2026-05-14

**Authors:** Ignas Lapeikis, Vincas Urbonas

**Affiliations:** 1 Faculty of Medicine, Lithuanian University of Health Sciences, 44307 Kaunas, Lithuania; ignas.lapeikis@stud.lsmu.lt; 2 Centre of Clinical Trials, National Cancer Institute, P. Baublio 3B, 08406 Vilnius, Lithuania

**Keywords:** melanoma, circulating cytokines, prognostic biomarkers

## Abstract

Cutaneous melanoma remains a highly lethal malignancy once metastatic. Current prognostic stratification relies primarily on staging and serum lactate dehydrogenase (LDH), which incompletely captures inter-patient biological heterogeneity. Increasing evidence highlights the importance of tumour–immune interactions in melanoma progression and response to therapy. This narrative review summarises and critically evaluates current evidence on circulating cytokines as prognostic and biologically informative biomarkers in melanoma, with particular emphasis on the immunotherapy era. Several circulating cytokines—most consistently interleukin-6 (IL-6) and interleukin-8 (IL-8)—are associated with adverse outcomes in advanced melanoma. However, baseline elevations predominantly reflect tumour burden and systemic inflammation, indicating prognostic rather than treatment-specific predictive value. In contrast, early on-treatment changes, particularly decreases in IL-8, may better capture evolving tumour–immune interactions during immune checkpoint inhibitor therapy. C-reactive protein (CRP), a downstream marker of IL-6 signalling, similarly reflects systemic inflammatory status and carries reproducible prognostic significance. Early circulating tumour DNA (ctDNA) dynamics demonstrate strong associations with response and survival and may provide complementary insight into tumour burden kinetics. Conversely, cytokines central to effective antitumour immunity, such as interferon-γ (IFN-γ), are more reliably characterised at the tumour transcriptional level than by circulating protein measurements. Circulating cytokines represent biologically meaningful but methodologically challenging biomarkers in melanoma. Their most realistic clinical role lies in complementing established prognostic factors within integrated biomarker frameworks rather than functioning as standalone tests. Standardisation of pre-analytical handling, assay platforms, and sampling time points, together with prospective validation, is essential before broader clinical implementation.

## 1. Introduction

Cutaneous melanoma represents a substantial and growing global health burden. Although it accounts for a relatively small proportion of all skin cancer diagnoses, melanoma is responsible for most of the skin cancer-related mortality worldwide, reflecting its pronounced metastatic potential and limited curability once disseminated disease develops [1]. Epidemiological data demonstrate a sustained increase in melanoma incidence over recent decades; however, recent analyses suggest that this trend is heterogeneous. In particular, incidence increases appear more pronounced among females, while remaining relatively stable among males in the last decade, and importantly, these changes have been accompanied by a decline in melanoma-specific mortality, likely reflecting earlier detection and therapeutic advances [2]. This rising incidence, combined with the substantial morbidity and mortality associated with advanced disease, continues to place significant pressure on healthcare systems.

Prognostic stratification in melanoma remains predominantly based on anatomical staging systems, most notably the American Joint Committee on Cancer (AJCC) classification, which incorporates tumour thickness, ulceration status, regional nodal involvement, and the presence of distant metastases [3]. While AJCC staging provides robust prognostic discrimination at the population level, it incompletely captures the marked biological heterogeneity observed among patients within the same stage. Serum lactate dehydrogenase (LDH) reflects complex tumour biology, including tissue hypoxia, metabolic reprogramming, and tumour aggressiveness, rather than serving as a direct surrogate of tumour burden alone. LDH should be interpreted as a composite marker reflecting hypoxic tumour microenvironment, metabolic activity, and aggressive disease biology, rather than a linear measure of tumour volume [4]. C-reactive protein (CRP), a readily available downstream marker of IL-6-driven systemic inflammation, has demonstrated reproducible prognostic associations in metastatic melanoma; however, similar to LDH, it largely reflects global inflammatory burden rather than tumour-specific immune dynamics. In contrast, circulating tumour DNA (ctDNA) has emerged as a dynamic biomarker capable of capturing real-time tumour burden kinetics and early treatment response, particularly in the context of immune checkpoint inhibition. Consequently, patients with similar AJCC stage and LDH levels frequently experience markedly divergent clinical courses, highlighting the limitations of current prognostic frameworks and the need for biomarkers that better reflect underlying disease biology. Melanoma is widely regarded as a prototypical immunogenic malignancy. Both spontaneous immune responses and therapeutic immune modulation play decisive roles in disease control, as evidenced by the durable clinical benefit achieved with immune checkpoint inhibitors targeting programmed cell death protein 1 (PD-1) and cytotoxic T-lymphocyte-associated antigen 4 (CTLA-4) [5,6]. These therapeutic advances have underscored the central importance of tumour–immune interactions in determining clinical outcomes. The melanoma tumour microenvironment (TME) is a highly dynamic ecosystem composed of malignant cells, immune infiltrates, stromal fibroblasts, endothelial cells, and extracellular matrix components. Interactions among these cellular and non-cellular elements critically shape tumour growth, immune evasion, metastatic dissemination, and response to systemic therapy [7].

Cytokines are key mediators of communication within the TME and between the tumour and the systemic immune system. Through pleiotropic and context-dependent effects, cytokines regulate immune cell recruitment, differentiation, activation, and suppression, as well as angiogenesis and tissue remodelling [8]. In melanoma, dysregulated cytokine networks contribute to chronic inflammation, immune dysfunction, and the establishment of immunosuppressive niches that facilitate tumour progression and resistance to therapy [9]. Beyond their mechanistic roles, many cytokines implicated in melanoma biology are detectable in peripheral blood, rendering them attractive candidates as minimally invasive biomarkers. In clinical practice, markers such as LDH and CRP primarily reflect systemic inflammatory and metabolic states rather than acting as direct surrogates of tumour burden. Moreover, emerging biomarkers such as ctDNA provide dynamic insight into tumour burden kinetics during immune checkpoint inhibition. Together, these circulating parameters offer a biologically informed but methodologically heterogeneous framework for risk stratification, underscoring the need for integrative models that distinguish prognostic inflammation from therapy-responsive immune modulation. Circulating cytokines may therefore provide integrative information capturing both tumour-intrinsic signalling and host immune responses. Several cytokines—including interleukin-6 (IL-6), interleukin-8 (IL-8), interleukin-10 (IL-10), tumour necrosis factor-α (TNF-α), interferon-γ (IFN-γ), and transforming growth factor-β (TGF-β)—have been investigated as potential prognostic biomarkers in melanoma, with multiple studies reporting associations with survival outcomes and response to systemic therapies [10,11,12,13]. However, findings across studies have been heterogeneous, influenced by differences in assay platforms, timing of sample collection, patient populations, and treatment eras. Moreover, interpretation of circulating cytokine levels is complicated by their pleiotropy, short circulating half-lives, and the spatially restricted nature of many cytokine-driven processes within the tumour microenvironment [14].

Given these biological and methodological challenges, a critical and mechanistically informed synthesis of the existing literature is required to distinguish cytokines that represent robust, biologically anchored prognostic markers from those whose apparent associations reflect confounding or analytical artefact. The aim of this review is therefore to summarise and critically evaluate current evidence linking circulating cytokine levels with patient outcomes in melanoma, with particular emphasis on the immunotherapy era. By integrating mechanistic insights with clinical data, we seek to clarify both the potential and the limitations of cytokines as prognostic biomarkers and to outline directions for their future integration into clinically meaningful, standardised biomarker frameworks.

It should be noted that cytokines represent one component of a broader repertoire of tumour-derived bioactive factors through which melanoma may influence systemic biology; these include neuroendocrine mediators, catecholamines, and melanogenic intermediates, whose immunomodulatory and homeostatic effects are subjects of active and growing investigation but fall outside the scope of the present cytokine-focused review.

## 2. A Clinical Framework for the Interpretation of Circulating Cytokines in Melanoma

To avoid conceptual ambiguity, circulating cytokines should be interpreted according to their clinical role and timing:

1. Prognostic biomarkers—Reflecting disease aggressiveness and natural history independent of therapy.
2. Predictive biomarkers—Identifying differential benefit from a specific treatment and requiring formal statistical interaction testing between biomarker status and treatment effect.
3. On-treatment monitoring biomarkers—Capturing early biological changes during therapy that may precede radiographic response or progression.

Importantly, the term state biomarker does not constitute a fourth clinical category but rather describes a biological characteristic. Many circulating cytokines—particularly IL-6 and IL-8—primarily reflect a systemic inflammatory or myeloid-dominant immune state. In the absence of appropriate interaction analyses, elevated baseline cytokines should not be misclassified as predictive biomarkers. Instead, they most often function as prognostic indicators of tumour burden, systemic inflammation, or host immune composition. Only when dynamic changes during therapy correlate specifically with treatment effect can a predictive interpretation be justified.

This distinction is essential to prevent conceptual inflation of biomarker claims and to ensure methodological rigour in translational melanoma research.

## 3. Cytokines in Melanoma Biology

Melanoma progression reflects dynamic tumour–host interactions within the tumour microenvironment (TME), where malignant cells coexist with immune and stromal components. Cytokines serve as measurable mediators of this interaction network, but their biological effects are highly context-dependent and may differ according to tumour burden, immune composition, and treatment exposure [7,15]. In advanced melanoma, dysregulated cytokine signalling does not represent a uniform pro-tumour signal; rather, it reflects heterogeneous inflammatory and immunological states that may variably influence immune escape and therapeutic response [9]. This biological variability complicates direct clinical interpretation of individual circulating cytokines and underscores the need for structured frameworks to contextualise their prognostic and predictive significance.

### 3.1. Pro-Inflammatory Versus Immunosuppressive Cytokines

Cytokines implicated in melanoma biology are often broadly categorised as pro-inflammatory or immunosuppressive; however, this dichotomy is inherently context dependent. Pro-inflammatory cytokines such as interleukin-6 (IL-6), interleukin-8 (IL-8), tumour necrosis factor-α (TNF-α), and interferon-γ (IFN-γ) are frequently upregulated in melanoma and reflect chronic inflammatory states associated with tumour progression [8,16]. While acute inflammatory signalling may promote antitumour immunity, sustained cytokine-driven inflammation can paradoxically support tumour growth by inducing angiogenesis, promoting genomic instability, and fostering immune exhaustion [17].

IL-6 and TNF-α exemplify this duality. Both cytokines activate survival and proliferation pathways in melanoma cells and promote recruitment of myeloid populations with immunosuppressive properties, thereby linking inflammation to immune evasion [18,19]. IL-8, a potent chemokine, drives neutrophil and myeloid-derived suppressor cell recruitment and is strongly associated with aggressive tumour behaviour and poor clinical outcomes [20]. In contrast, IFN-γ plays a critical role in antitumour immunity by enhancing antigen presentation and effector T-cell recruitment, yet chronic IFN-γ signalling can induce adaptive resistance mechanisms, including upregulation of immune checkpoint ligands [21].

Immunosuppressive cytokines, particularly interleukin-10 (IL-10) and transforming growth factor-β (TGF-β), contribute to immune escape by inhibiting dendritic cell maturation, suppressing cytotoxic T-cell function, and promoting regulatory immune cell populations [22,23]. TGF-β additionally exerts profound effects on stromal remodelling, leading to immune exclusion phenotypes characterised by impaired T-cell infiltration into tumour nests [24]. The balance between pro-inflammatory and immunosuppressive cytokines within the TME, therefore, critically determines the trajectory of melanoma progression and response to therapy.

### 3.2. Key Cytokine-Regulated Signalling Pathways

The biological effects of cytokines in melanoma converge on a limited number of intracellular signalling pathways, most notably nuclear factor kappa B (NF-κB), Janus kinase/signal transducer and activator of transcription (JAK/STAT), and interferon signalling cascades. These pathways integrate inflammatory cues with oncogenic signals to regulate tumour cell behaviour and immune interactions.

NF-κB is a central mediator of inflammation-induced tumour promotion. In melanoma, NF-κB activation drives transcription of genes involved in cell survival, proliferation, angiogenesis, and resistance to apoptosis [25]. Chronic NF-κB signalling promotes a pro-tumorigenic inflammatory milieu and supports the expression of cytokines and chemokines that recruit immunosuppressive myeloid cells [26].

JAK/STAT signalling, particularly via STAT3, represents a dominant axis linking cytokine signalling to melanoma progression. IL-6-mediated STAT3 activation enhances tumour cell survival and proliferation while simultaneously suppressing antitumour immunity through expansion and stabilisation of myeloid-derived suppressor cells [18,27]. Persistent STAT3 activation has been observed in melanoma and is associated with poor prognosis and resistance to immune-mediated control [28].

Interferon signalling, predominantly mediated by IFN-γ, is essential for effective antitumour immune responses. IFN-γ induces expression of major histocompatibility complex molecules and components of the antigen-processing machinery, thereby enhancing tumour immunogenicity [16]. However, prolonged interferon signalling can also induce expression of immune inhibitory molecules such as PD-L1 and contribute to immune exhaustion and acquired resistance to immune checkpoint blockade [29]. This dual role underscores the complexity of interpreting cytokine-driven signalling in melanoma.

### 3.3. Impact on Tumour Growth, Angiogenesis, and Immune Evasion

Through activation of these pathways, cytokines exert direct and indirect effects on melanoma growth and dissemination. Pro-inflammatory cytokines promote angiogenesis primarily through induction of vascular endothelial growth factor (VEGF), the principal regulator of tumour neovascularisation, along with other pro-angiogenic mediators that facilitate tumour expansion and metastatic spread [30]. IL-8 contributes to tumour-associated angiogenesis and vascular remodelling through CXCR1/2-mediated endothelial activation and recruitment of pro-angiogenic myeloid cells, thereby amplifying VEGF-driven pathways rather than acting as the dominant angiogenic driver [11,31,32]. Cytokines also shape immune evasion strategies by modulating immune cell composition and function within the TME. IL-6, IL-8, and TNF-α promote accumulation of immunosuppressive myeloid populations, while IL-10 and TGF-β inhibit effector T-cell activity and promote regulatory T-cell differentiation [19,33]. These effects collectively dampen antitumour immune surveillance and undermine the efficacy of immunotherapeutic interventions.

Importantly, many of these cytokine-driven processes are spatially restricted to the tumour microenvironment and may not be fully captured by circulating cytokine levels. This spatial biology partially explains the inconsistent performance of some cytokines as systemic biomarkers despite their clear mechanistic relevance in melanoma progression [34]. Nonetheless, understanding these biological roles is essential for interpreting associations between circulating cytokines and clinical outcomes.

## 4. Evidence for Individual Cytokines as Prognostic Markers

### 4.1. Interleukin-6 (IL-6)

Among circulating cytokines evaluated in melanoma, interleukin-6 (IL-6) has demonstrated one of the most consistent associations with adverse clinical outcomes. Elevated baseline serum IL-6 levels have been linked to shorter overall survival (OS) and progression-free survival (PFS) across multiple independent cohorts of patients with advanced melanoma [12,13,35]. In a well-characterised cohort, Fridman et al. reported that high IL-6 levels were independently associated with inferior OS after adjustment for stage and performance status, highlighting its prognostic relevance beyond tumour burden alone [34]. Subsequent studies, including analyses conducted in the immunotherapy era, have corroborated these findings, although effect sizes are attenuated when serum LDH and radiographic tumour burden are rigorously controlled [12]. Collectively, these data support IL-6 primarily as a prognostic biomarker reflecting systemic inflammatory activation and aggressive disease biology.

IL-6 signalling induces hepatic production of CRP, a routinely measured downstream marker of systemic inflammation. Circulating IL-6 and CRP levels frequently correlate in patients with advanced melanoma, and elevated CRP has similarly been associated with inferior survival outcomes. However, CRP reflects downstream inflammatory activation rather than pathway-specific immune signalling, positioning IL-6 as a more mechanistically proximal indicator of tumour–host interaction while acknowledging the clinical practicality and standardisation advantages of CRP measurement.

The biological plausibility of IL-6 as a prognostic marker is supported by its central role in activating the JAK/STAT3 pathway. IL-6-mediated STAT3 activation promotes melanoma cell survival, proliferation, and resistance to apoptosis, while also facilitating expansion and stabilisation of myeloid-derived suppressor cells that inhibit antitumour T-cell responses [19,27,32]. These tumour-intrinsic and immune-mediated effects provide a mechanistic basis for the association between elevated circulating IL-6 and adverse outcomes.

Importantly, translational exploration of IL-6 pathway modulation has been undertaken in the immunotherapy setting. Selective IL-6 receptor inhibition has been used to manage immune-related adverse events during immune checkpoint inhibitor therapy, demonstrating effective suppression of systemic inflammation [35]. While these data do not establish IL-6 blockade as an antitumour strategy, they underscore the clinical manipulability and biological relevance of the IL-6 axis in patients receiving immune checkpoint inhibitors.

Critically, IL-6 is a non-specific inflammatory cytokine and may be elevated in a range of non-malignant conditions, including infection and systemic inflammatory states such as COVID-19. Therefore, interpretation of IL-6 levels requires careful clinical context, particularly in patients with concomitant inflammatory or infectious conditions.

### 4.2. Interleukin-8 (IL-8, CXCL8)

Interleukin-8 (IL-8) has emerged as one of the most robust circulating prognostic biomarkers in melanoma, particularly in patients treated with immune checkpoint inhibitors. Elevated baseline IL-8 levels have been consistently associated with aggressive disease features and inferior survival outcomes [13,35]. In a landmark study by Sanmamed et al., high serum IL-8 levels were shown to predict resistance to PD-1 blockade and reduced OS, independent of baseline tumour burden, across melanoma and other solid tumour cohorts [36].

Mechanistically, IL-8 promotes recruitment of neutrophils and polymorphonuclear myeloid-derived suppressor cells through CXCR1/2 signalling, thereby fostering an immunosuppressive tumour microenvironment [37,38]. In addition, IL-8 exerts potent pro-angiogenic effects, stimulating endothelial cell proliferation and vascular remodelling, which facilitate tumour growth and metastatic dissemination [31]. Notably, dynamic changes in IL-8 levels during treatment have been shown to correlate with early response or progression under immunotherapy, underscoring its potential utility as both a prognostic and on-treatment biomarker [39].

### 4.3. Interleukin-10 (IL-10)

Interleukin-10 (IL-10) is a key immunosuppressive cytokine that inhibits antigen presentation and effector T-cell activation. Several studies have reported that elevated circulating IL-10 levels are associated with worse survival outcomes in melanoma, consistent with its role in dampening antitumour immune responses [40,41]. Early clinical investigations identified IL-10 as part of an immunosuppressive cytokine profile linked to advanced disease and poor prognosis [42].

However, the prognostic performance of IL-10 has been inconsistent across cohorts. In several analyses, IL-10 lost independent prognostic significance after adjustment for disease stage or tumour burden [43]. Biologically, IL-10 exerts dual effects, as it may also limit excessive inflammation and tissue damage, potentially conferring context-dependent protective roles [44,45]. This functional duality likely contributes to the heterogeneity of reported associations and limits the clinical applicability of IL-10 as a standalone prognostic biomarker.

### 4.4. Tumour Necrosis Factor-α (TNF-α)

Tumour necrosis factor-α (TNF-α) is a central mediator of inflammation with pleiotropic effects on tumour biology and immune regulation. In melanoma, elevated circulating TNF-α levels have been associated with poor survival in some cohorts, reflecting chronic inflammatory states and activation of tumour-promoting NF-κB signalling pathways [9,16]. However, other studies have failed to demonstrate a consistent or independent prognostic association [16].

The limited prognostic utility of TNF-α likely reflects its short half-life, sensitivity to non-malignant inflammatory stimuli, and context-dependent biological effects. While TNF-α has gained increasing attention as a mediator of immune-related adverse events during immune checkpoint blockade and as a potential therapeutic target to mitigate toxicity, current evidence does not support its use as a reliable prognostic biomarker in melanoma [46].

### 4.5. Interferon-γ (IFN-γ)

Interferon-γ (IFN-γ) is a hallmark cytokine of effective antitumour immunity, promoting antigen presentation, T-cell recruitment, and cytotoxic effector function. Higher levels of IFN-γ activity are generally associated with immune-inflamed tumour phenotypes and improved response to immune checkpoint inhibitors [47]. Indeed, tumour-level interferon-related gene expression signatures are among the most robust predictors of response to PD-1 blockade in melanoma [48,49].

In contrast, circulating IFN-γ levels have shown inconsistent associations with clinical outcomes. Serum IFN-γ concentrations are typically low, transient, and spatially uncoupled from tumour microenvironment activity, limiting their prognostic value [50]. As a result, IFN-γ is best regarded as a mechanistically central but poorly captured circulating biomarker, with greater utility at the tissue or pathway level rather than as a serum-based prognostic marker.

### 4.6. Transforming Growth Factor-β (TGF-β)

Transforming growth factor-β (TGF-β) is a potent immunosuppressive cytokine that promotes immune evasion, stromal remodelling, and metastatic progression in melanoma. Elevated TGF-β signalling has been associated with immune-excluded tumour phenotypes and resistance to immune checkpoint blockade [24,51,52]. Several studies have reported associations between higher circulating TGF-β levels and poor prognosis, although findings are inconsistent [53,54].

The prognostic performance of circulating TGF-β is limited by significant pre-analytical variability and its predominantly localised activity within the tumour microenvironment. Consequently, TGF-β pathway activity is more accurately captured through tissue-based signatures rather than serum measurements [55].

### 4.7. Emerging Cytokines

Emerging evidence suggests that additional cytokines and chemokines, including CXCL9, CXCL10, granulocyte–macrophage colony-stimulating factor (GM-CSF), and members of the IL-17 family, may hold prognostic or predictive relevance in melanoma. CXCL9 and CXCL10, interferon-inducible chemokines, have been associated with T-cell-inflamed tumour microenvironments and favourable responses to immunotherapy [56,57]. GM-CSF demonstrates context-dependent effects in melanoma, with evidence suggesting both immunostimulatory and immunosuppressive roles. Preclinical and clinical data indicate that GM-CSF may, under certain conditions, promote expansion of immunosuppressive myeloid populations, highlighting the complexity of its biological role [58,59]. Cytokines of the IL-17 family have been implicated in inflammation-driven tumour progression, but clinical data in melanoma remain limited and heterogeneous [60,61]. Overall, these mediators require further validation in large, prospective cohorts before clinical application can be considered.

Taken together, the available evidence indicates that individual circulating cytokines rarely function as isolated, therapy-specific determinants of outcome. Instead, they reflect overlapping biological processes—including systemic inflammation, myeloid activation, immune suppression, and stromal remodelling—that variably influence melanoma progression and response to immunotherapy. Table 1 summarises the dominant biological roles, mechanistic pathways, and clinically relevant interpretations of key circulating cytokines in melanoma. Importantly, this synthesis emphasises that the prognostic and predictive value of cytokines is highly context-dependent and should be interpreted within integrated biological and clinical frameworks rather than as standalone biomarkers.

## 5. Cytokines in the Era of Immunotherapy

In the era of immune checkpoint inhibition, treatment outcomes in advanced melanoma are characterised by marked biological heterogeneity. While PD-1/PD-L1 and CTLA-4 blockade achieve durable responses in a subset of patients, primary resistance and early progression remain common despite adequate drug exposure [5,6]. This variability has intensified interest in circulating biomarkers capable of reflecting tumour–immune dynamics and identifying early resistance phenotypes.

This clinical heterogeneity has driven intense interest in biomarkers that reflect the evolving tumour–host equilibrium under immunotherapy and circulating cytokines—because they are readily serially measurable—have been investigated both as baseline risk stratifiers and as dynamic pharmacodynamic readouts [11,36]. Importantly, the biological meaning of cytokines in this context differs by time point: baseline levels primarily encode pre-existing inflammatory and immune states, whereas on-treatment changes can reflect early biological response, emerging resistance programmes, or immune toxicity [35,67].

A consistent pattern across melanoma cohorts is that baseline elevations of cytokines linked to myeloid inflammation (particularly IL-6 and IL-8) associate with inferior outcomes on PD-1-based therapy, plausibly reflecting a suppressive, myeloid-dominant immune architecture that constrains effective T-cell reinvigoration [11,36]. IL-8 is the clearest example of a cytokine with both mechanistic anchoring and clinically useful dynamics under ICI. In a seminal translational analysis, changes in serum IL-8 during checkpoint blockade tracked clinical benefit and preceded radiographic response/progression in melanoma and other tumour types, supporting IL-8 as a real-time biomarker of tumour–myeloid inflammatory momentum rather than a static correlate of disease burden [36]. This observation has practical implications: a falling IL-8 trajectory early after therapy initiation is biologically consistent with attenuation of tumour-promoting myeloid inflammation, while rising IL-8 suggests ongoing recruitment/activation of neutrophil-lineage suppressor populations and angiogenic signalling that frequently co-segregate with resistance [36,37]. Beyond IL-6 and IL-8, other cytokines such as TNF-α further illustrate the context-dependent complexity of biomarker interpretation in the immunotherapy setting.

### TNF-α in the Context of Immunotherapy and Biomarker Interpretation

Tumour necrosis factor-alpha (TNF-α) represents a prototypical example of a context-dependent cytokine in melanoma immunotherapy. TNF-α signalling contributes to immune activation, T-cell trafficking, and inflammatory amplification, yet it is also centrally involved in the pathogenesis of immune-related adverse events (irAEs). In clinical practice, anti-TNF agents such as infliximab are routinely administered for steroid-refractory ICI-induced colitis and other severe toxicities. Retrospective melanoma cohorts indicate that short-term TNF blockade for irAE management does not consistently compromise objective response rates or overall survival in patients receiving immune checkpoint inhibitors [64,65,68].

These observations underscore the dualistic nature of TNF biology: while TNF contributes to antitumour immune activity, its systemic elevation may also reflect inflammatory toxicity rather than effective immune-mediated tumour control. Consequently, circulating TNF levels are unlikely to function as straightforward predictive biomarkers of ICI efficacy. Instead, TNF may better represent a state marker of systemic inflammatory activation, the clinical significance of which depends on timing, context, and concurrent immunosuppressive exposure. This complexity reinforces the rationale for integrative, multi-parameter models—rather than reliance on single cytokines—to interpret tumour–immune dynamics during immunotherapy.

## 6. Timing of Cytokine Assessment

Baseline versus on-treatment cytokine profiling should be interpreted through this biological lens. Baseline cytokines often capture an “inflammatory set point” shaped by tumour burden, necrosis, and host comorbidity; therefore, they may enrich for poor-prognosis biology without necessarily being treatment-specific predictors [69]. By contrast, early on-treatment cytokine dynamics can be closer to causal biology, because they report how the tumour–immune system responds to PD-1/CTLA-4 perturbation in vivo [36,67]. A key limitation, however, is compartmentalisation: cytokines central to effective antitumour immunity (e.g., IFN-γ) exert much of their clinically relevant activity within the TME, so circulating concentrations may fail to reflect tissue-level pathway activation even when tumour interferon signalling is robust [29,70]. Accordingly, tumour-level interferon-related transcriptional programmes have been repeatedly associated with response to PD-1 blockade, whereas serum IFN-γ has been a comparatively unstable and inconsistent metric across cohorts [66,71].

Cytokines also intersect with toxicity biology. Immune-related adverse events (irAEs) are frequent in melanoma under PD-1 and CTLA-4 blockade and reflect systemic immune activation that is not perfectly coupled to antitumour efficacy [72]. In this setting, circulating cytokines are attractive because they may identify patients at risk for clinically relevant toxicity before severe manifestations occur. A focused melanoma study demonstrated that circulating cytokine patterns could predict immune-related toxicity during anti-PD-1-based immunotherapy, supporting the concept that systemic cytokine networks encode host inflammatory liability under checkpoint blockade [73]. Clinically, this matters because an early cytokine “toxicity signature” could inform monitoring intensity, supportive care, and potentially early intervention strategies—without requiring tumour tissue [73]. At the same time, cytokines implicated in irAEs are often nonspecific markers of inflammation and may be influenced by infection, baseline autoimmunity, or concomitant medications, necessitating careful clinical adjudication and prospective validation before any decision-support use [73,74].

These considerations argue strongly against single-cytokine decision-making and in favour of cytokine panels interpreted as mechanistically coherent modules. Multiplex cytokine profiling can capture coordinated inflammatory states that individual analytes cannot, improving signal-to-noise and reducing vulnerability to assay variability, biological fluctuation, and pleiotropy [75]. In practice, the most promising use-cases are: (i) baseline risk stratification for primary resistance (myeloid/inflammatory modules dominated by IL-6/IL-8), (ii) early on-treatment monitoring for biological response versus imminent progression (dynamic IL-8 and related chemokines), and (iii) toxicity risk enrichment (cytokine patterns linked to systemic immune activation) [11,35,74]. However, translation requires discipline: panel composition must be hypothesis-driven; sampling time points must be standardised; and models must be prospectively validated against prespecified clinical endpoints in multi-centre cohorts, ideally integrated with orthogonal biomarkers such as ctDNA kinetics, immune cell phenotyping, and tumour transcriptional/spatial profiling [67,75,76].

In summary, in the immunotherapy era, cytokines are best conceptualised as state biomarkers rather than definitive predictors. Baseline cytokines often reflect a pre-treatment inflammatory architecture associated with poor prognosis, while early on-treatment changes—most convincingly demonstrated for IL-8—can provide actionable insight into evolving tumour–immune dynamics [11,35]. The realistic clinical future is not “a cytokine test that replaces staging,” but integrated models in which cytokine modules complement tumour- and blood-based biomarkers to guide early treatment adaptation and toxicity surveillance [75,76].

## 7. Conceptual Framework: How Should Cytokines Be Interpreted?

Melanoma prognosis in the metastatic setting continues to rely primarily on disease stage and serum lactate dehydrogenase (LDH). Elevated LDH remains one of the strongest adverse prognostic markers and is incorporated into staging systems and contemporary clinical guidelines [3,77,78].

Systemic inflammatory markers such as the neutrophil-to-lymphocyte ratio (NLR) have demonstrated reproducible associations with survival in melanoma and other solid tumours, reflecting host immune status and myeloid-driven inflammation [79,80].

Cytokines, particularly IL-6, IL-8, and IL-10, represent biologically plausible mediators of melanoma progression and immune escape. Elevated IL-8 has been associated with poorer overall survival and inferior outcomes in patients receiving anti-PD-1 therapy [11,63]. IL-6 has been linked to tumour burden, systemic inflammation, and resistance mechanisms mediated through STAT3 signalling [79]. IL-10, an immunosuppressive cytokine, has been correlated with impaired antitumour T-cell responses and adverse prognosis across multiple cohorts [62].

Importantly, baseline cytokine levels alone may not fully capture therapeutic dynamics. Emerging evidence suggests that early changes in circulating tumour DNA (ctDNA) during ICI therapy strongly correlate with radiographic response and survival, often preceding imaging findings [81,82,83,84]. Similarly, decreases in IL-8 during treatment have been associated with improved clinical outcomes [62].

The MEL-IMMUNE model, therefore, integrates:

- Established clinical prognostic variables (LDH, performance status, and metastatic pattern);
- Systemic immune-inflammatory markers (NLR);
- Circulating cytokines reflecting tumour–immune crosstalk (IL-6, IL-8, and IL-10);
- Early on-treatment tumour burden dynamics (ctDNA kinetics).

This composite framework is conceptually analogous to established risk scores in other malignancies but tailored to the immunobiology of melanoma. Rather than replacing conventional staging, MEL-IMMUNE aims to refine biological risk stratification and identify early resistance phenotypes in patients receiving ICI.

Multivariate serum-based models have previously demonstrated prognostic and predictive associations in melanoma treated with PD-1 blockade; for example, Weber et al. identified a serum protein signature associated with survival outcomes following anti-PD-1 therapy, underscoring the potential value of integrated biomarker approaches rather than isolated analytes [85].

**Figure 1:**
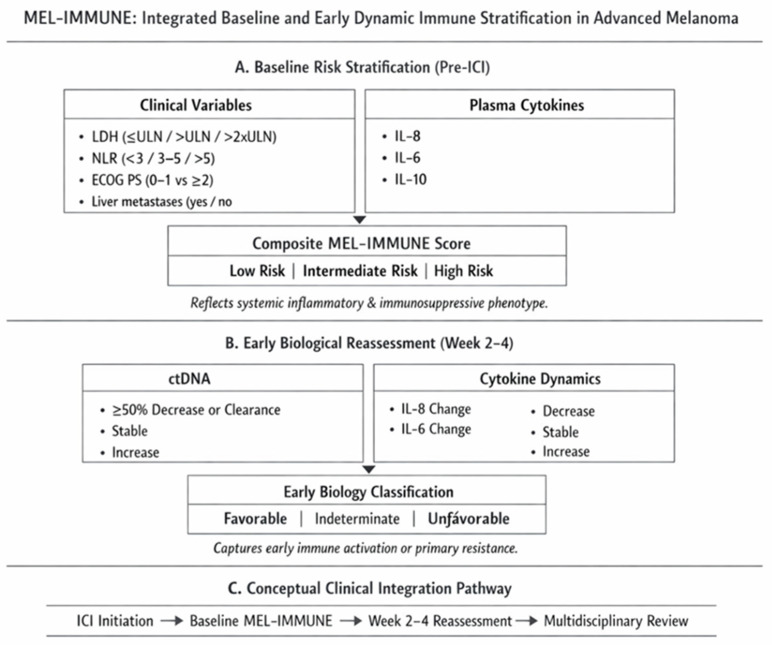
Conceptual architecture of the MEL-IMMUNE risk model.

The proposed MEL-IMMUNE framework should be regarded as a conceptual and hypothesis-generating model rather than a validated clinical tool. The assignment of point values is not derived from multivariable statistical modelling and therefore does not reflect independent prognostic weighting. Future development of such models should rely on rigorously derived coefficients from large, prospective datasets, ensuring inclusion of only independent variables. Additionally, the selection of variables—including metastatic patterns and patient characteristics—requires systematic evaluation, as factors such as brain metastases and sex-specific differences may influence outcomes (Table 2, Figure 1).

MEL-IMMUNE integrates established clinical prognostic variables (LDH, NLR, ECOG, and metastatic pattern) with circulating cytokines (IL-6, IL-8, and IL-10) and early ctDNA kinetics to refine biological risk stratification in advanced melanoma treated with immune checkpoint inhibitors. The model consists of a baseline composite risk score and an early on-treatment dynamic component reflecting immune activation or resistance. MEL-IMMUNE is intended as a biologically informed stratification tool requiring prospective validation before clinical implementation.

## 8. Challenges and Limitations

Despite growing interest in circulating cytokines as prognostic biomarkers in melanoma, several methodological and biological limitations have thus far precluded their routine clinical implementation. A major challenge arises from substantial assay heterogeneity across studies. Cytokine measurements have been performed using single-analyte enzyme-linked immunosorbent assays (ELISAs) as well as multiplex bead-based platforms, which differ markedly in sensitivity, dynamic range, cross-reactivity, and susceptibility to matrix effects [75,86]. These technical differences can result in systematic variation in absolute cytokine concentrations, limiting comparability across cohorts and complicating attempts at external validation.

Pre-analytical factors further contribute to variability. Serum and plasma are both commonly used for cytokine assessment, yet cytokine concentrations may differ significantly between these matrices due to clotting-related cytokine release, platelet activation, and proteolytic degradation [87,88,89]. Inadequate standardisation of sample collection, processing, and storage conditions introduces additional noise, particularly for cytokines with short half-lives or low circulating concentrations, such as IFN-γ. Together, these factors undermine reproducibility and limit the transferability of proposed cut-off values across studies.

The timing of cytokine measurement represents another critical limitation. Many studies rely on baseline cytokine levels, which may primarily reflect tumour burden, necrosis, or systemic inflammation rather than biologically meaningful immune states relevant to treatment response [90]. In contrast, on-treatment cytokine dynamics may better capture evolving tumour–immune interactions, particularly in the context of immune checkpoint blockade [35]. However, longitudinal sampling is inconsistently performed, and time points vary widely between studies, making it difficult to define optimal windows for prognostic or predictive assessment.

Lack of standardisation in the cut-off definition further complicates interpretation. Cytokine thresholds have been defined using medians, tertiles, quartiles, or receiver operating characteristic (ROC)-derived cut-offs, often without independent validation [91,92,93]. As a result, reported associations may be cohort-specific and vulnerable to overfitting. This issue is particularly problematic when cytokines are evaluated individually rather than as part of multivariable or multicytokine models that account for biological interdependence and reduce the impact of arbitrary dichotomisation.

Confounding clinical factors also represent a major challenge. Cytokine levels are strongly influenced by disease stage, tumour burden, and systemic inflammation, all of which are themselves associated with prognosis [94]. Serum LDH correlates with both tumour burden and inflammatory cytokines, raising concerns that some reported prognostic associations may reflect residual confounding rather than independent biological effects [95,96]. Additionally, the treatment era is a critical modifier: studies conducted prior to the widespread adoption of immune checkpoint inhibitors may not be directly comparable to contemporary cohorts, in which immune modulation fundamentally alters cytokine dynamics and their clinical interpretation [97].

Finally, the spatial biology of cytokine signalling imposes intrinsic limitations on circulating measurements. Many cytokines exert their most relevant biological effects locally within the tumour microenvironment, where concentration gradients and cell–cell interactions cannot be inferred from peripheral blood levels [7]. Consequently, circulating cytokines may function better as indirect markers of global inflammatory or immune states rather than precise surrogates of intratumoral signalling activity.

Taken together, these challenges underscore the need for rigorous standardisation of assay methodology, harmonised sampling strategies, and careful multivariable modelling in future studies [98]. Without such measures, the clinical translation of cytokines as prognostic biomarkers in melanoma will remain limited, despite compelling mechanistic rationale and accumulating associative evidence.

## 9. Future Directions

The future clinical utility of cytokines as prognostic biomarkers in melanoma will depend on moving beyond single-analyte measurements toward integrated, biologically informed biomarker frameworks. Given the pleiotropic and highly interconnected nature of cytokine signalling, isolated assessment of individual cytokines is unlikely to adequately capture the complexity of tumour–immune interactions that govern disease progression and therapeutic response. Instead, multiplex cytokine panels that reflect coordinated inflammatory and immunosuppressive programmes represent a more rational approach [99,100].

Recent advances in multiplex immunoassay technologies and high-throughput proteomics now allow for simultaneous quantification of dozens of cytokines from limited sample volumes with acceptable analytical performance. When combined with robust bioinformatic modelling, such approaches can identify cytokine signatures that outperform individual markers in prognostic and predictive accuracy [101,102]. Importantly, cytokine profiling should not be pursued in isolation but integrated with complementary biomarkers that capture distinct dimensions of tumour biology. Circulating tumour DNA (ctDNA) provides a quantitative measure of tumour burden and clonal dynamics, while tumour-infiltrating lymphocytes (TILs) and immune gene expression signatures reflect local immune activation [103,104]. Spatial transcriptomic technologies further enable resolution of cytokine-driven signalling within defined tumour niches, addressing a key limitation of peripheral blood-based measurements [105].

Prospective validation in large, multicentre cohorts is essential to establish the clinical relevance of cytokine-based biomarkers. Many existing studies are retrospective, underpowered, or conducted within narrowly defined patient populations, limiting generalisability. Future trials should incorporate pre-specified biomarker hypotheses, harmonised sampling schedules, and standardised assay platforms to enable meaningful cross-study comparisons [106]. Importantly, cytokine analyses should be embedded within contemporary clinical trials of immune checkpoint inhibitors and combination regimens, ensuring relevance to current treatment paradigms [107]. Only through such rigorously designed prospective studies can the independent prognostic value of cytokine signatures be distinguished from confounding effects related to tumour burden, disease stage, or treatment exposure.

Standardisation of reporting and consensus on analytical cut-offs represent additional prerequisites for clinical translation. At present, variability in assay platforms, sample matrices, and statistical methodologies has resulted in inconsistent cut-off definitions and limited reproducibility across cohorts. International efforts to harmonise biomarker reporting standards, including transparent documentation of pre-analytical variables and validation procedures, are critical to overcoming these barriers [108]. Rather than relying on arbitrary dichotomisation, future studies should prioritise continuous modelling approaches or externally validated thresholds derived from independent training and validation cohorts [109,110,111,112].

Ultimately, the integration of cytokine profiling into multimodal biomarker strategies holds promise for refining prognostic stratification in melanoma. By combining systemic inflammatory signals with tumour-intrinsic and microenvironmental features, cytokine-based panels may contribute to more precise risk assessment, patient selection, and treatment sequencing in the immunotherapy era. However, realisation of this potential will require coordinated methodological standardisation, prospective validation, and close alignment between biological insight and clinical trial design.

## 10. Conclusions

Accumulating evidence over the past decade indicates that circulating cytokines capture biologically and clinically relevant aspects of melanoma progression. Among the mediators studied to date, interleukin-6 (IL-6), interleukin-8 (IL-8), and interleukin-10 (IL-10) have shown the most consistent associations with adverse clinical outcomes across independent cohorts, particularly in patients with advanced disease. These associations are biologically plausible, reflecting cytokine-driven activation of tumour-promoting inflammatory pathways, recruitment of immunosuppressive myeloid populations, and attenuation of effective antitumour immune responses. In contrast, cytokines such as IFN-γ and TGF-β appear to exert context-dependent effects that are more accurately captured at the tissue or pathway level rather than through isolated circulating measurements.

Despite compelling mechanistic rationale and repeated associative signals, cytokines have not yet achieved clinical utility as standalone prognostic biomarkers in melanoma. Methodological heterogeneity, including variability in assay platforms, sample matrices, timing of measurement, and cut-off definitions, has limited reproducibility and cross-study comparability. In addition, confounding by tumour burden, disease stage, and treatment context—particularly in the rapidly evolving immunotherapy era—remains a major challenge to establishing independent prognostic value.

Future clinical translation will require rigorously designed prospective studies incorporating harmonised sampling protocols, standardised analytical platforms, and predefined statistical frameworks. Rather than focusing on individual cytokines, integrated cytokine panels combined with complementary biomarkers such as ctDNA and immune contexture metrics are more likely to provide robust and clinically actionable prognostic information. Until such evidence is generated and validated, circulating cytokines should be regarded as biologically informative research tools rather than ready-to-use clinical biomarkers.

## Figures and Tables

**Table 1:** Biological role and clinical relevance of key circulating cytokines in melanoma.

Cytokine	Prognostic Association	Dominant Biological Role	Key Mechanisms/Pathways	Clinical Interpretation	Key References
IL-6	Consistently adverse (OS, PFS)	Pro-tumour inflammation; systemic immune suppression	JAK/STAT3 activation; MDSCs (myeloid-derived suppressor cells) expansion; angiogenesis	Robust negative prognostic marker; reflects tumour burden and inflammatory resistance biology	[18,19,62]
IL-8 (CXCL8)	Strongly adverse; dynamic	Myeloid recruitment; angiogenesis	CXCR1/2–NF-κB; neutrophil/MDSC trafficking	Best validated circulating cytokine for on-treatment monitoring and early resistance	[20,36,63]
IL-10	Context-dependent; often adverse	Immunosuppression; tolerance induction	STAT3-mediated APC and T-cell inhibition	Limited standalone prognostic value; contributes to suppressive cytokine modules	[22,45,54]
TNF-α	Variable; context-dependent	Chronic inflammation; immune modulation	TNFR1/2–NF-κB signalling; T-cell exhaustion	Poor standalone prognostic utility; more relevant to toxicity and resistance biology	[46,64,65]
IFN-γ	Favourable at tissue/pathway level	Th1 immunity; antigen presentation	JAK/STAT1; CXCL9/10 induction	Strong predictor of immunotherapy response in tumour, poorly captured in serum	[48,56,66]
TGF-β	Adverse (contextual)	Immune exclusion; stromal remodelling	SMAD2/3 signalling; T-cell suppression	Central resistance pathway; serum levels variable, tissue signatures preferred	[24,53,55]

**Table 2:** MEL-IMMUNE score: Composite baseline and early dynamic risk model in advanced melanoma.

**A. Baseline MEL-IMMUNE Score (Pre-ICI)**				
**Domain**		**Variable**	**Criteria**	**Points**
**Tumour burden**		LDH	≤ULN *	0
			>ULN	+2
			>2 × ULN	+3
**Systemic inflammation**		NLR	<3	0
			3–5	+1
			>5	+2
**Clinical status**		ECOG PS **	0–1	0
			≥2	+1
**Metastatic pattern**		Liver metastases	No	0
			Yes	+1
**Immune mediators (plasma)**		IL-8	Above threshold	+2
		IL-6	Above threshold	+1
		IL-10	Above threshold	+1
**Baseline Risk Categories**				
**Total Score**	**Risk Group**	**Expected Biology**		
0–2	Low	Limited systemic inflammatory activation		
3–5	Intermediate	Mixed immune-inflammatory phenotype		
≥6	High	Myeloid-driven/immunosuppressive systemic state		
**B. Early MEL-IMMUNE Dynamic Score (Week 2–4)**				
**Variable**	**Change from Baseline**	**Points**		
ctDNA	≥50% decrease or clearance	−3		
IL-8	≥30–50% decrease	−2		
IL-6	≥30% decrease	−1		
IL-8	Any significant increase	+1		
IL-6	Any significant increase	+1		
**Early Biology Classification**				
**Dynamic Score**	**Interpretation**	Suggested Clinical Consideration ***		
≤−3	Favourable early biology	Continue current strategy		
−2 to +1	Indeterminate	Standard imaging schedule		
≥+2	Unfavourable early biology	Consider early imaging/trial discussion		

* ULN—Upper Limit of Normal; ** ECOG—Eastern Cooperative Oncology Group Performance Status. *** Exploratory; requires prospective validation.

## Data Availability

All available data were included in this published review.
